# Zinc oxide nanoparticles enhanced rice yield, quality, and zinc content of edible grain fraction synergistically

**DOI:** 10.3389/fpls.2023.1196201

**Published:** 2023-08-18

**Authors:** Kailiang Mi, Xijun Yuan, Qianyue Wang, Canping Dun, Rui Wang, Shuo Yang, Yanju Yang, Hongcheng Zhang, Haipeng Zhang

**Affiliations:** Jiangsu Key Laboratory of Crop Cultivation and Physiology/Co-Innovation Center for Modern Production Technology of Grain Crops/Innovation Center of Rice Cultivation Technology in Yangtze Valley, Research Institute of Rice Industrial Engineering Technology, Yangzhou University, Yangzhou, China

**Keywords:** zinc oxide nanoparticles, soil application, rice quality, zinc distribution, zinc bioavailability

## Abstract

Zinc oxide nanoparticles (ZnO NPs) have been widely used in agriculture as a new type of Zn fertilizer, and many studies were conducted to evaluate the effect of ZnO NPs on plant growth. However, there are relatively few studies on the effects of application methods and appropriate dosages of ZnO NPs on rice yield, quality, grain Zn content, and distribution. Therefore, in the 2019 and 2020, field trials were conducted with six ZnO NPs basal application dosages of no ZnO NPs, 3.75 kg hm^−2^, 7.5 kg hm^−2^, 15 kg hm^−2^, 30 kg hm^−2^, and 60 kg hm^−2^, and the effects of ZnO NPs application on rice yield, quality, grain Zn content, and distribution were investigated. The results demonstrated that applying ZnO NPs in Zn-deficient soils (available Zn < 1.0 mg kg^−1^) increased rice grain yield by 3.24%–4.86% and 3.51%–5.12% in 2019 and 2020, respectively. In addition, ZnO NPs improved the quality of rice by increasing the head milling rate, reducing chalky grain percentage, and increasing the taste value and breakdown of rice. In terms of Zn accumulation in rice, ZnO NPs application significantly increased the Zn content in both milled rice and brown rice, compared with no Zn treatment, in 2019 and 2020, Zn content in milled rice significantly increased by 20.46%–41.09% and 18.11%–38.84%, respectively, and in brown rice significantly increased by 25.78%–48.30% and 20.86%–42.00%, respectively. However, the Zn fertilizer utilization gradually decreased with increasing ZnO NPs application dosage. From the perspective of yield, rice quality, Zn fertilizer utilization, and Zn accumulation, basal application of 7.5 kg–30 kg hm^−2^ ZnO NPs is beneficial for rice yield and quality improvement and rice Zn accumulation. This study effectively demonstrated that ZnO NPs could be a potential high‐performed fertilizer for enhancing rice yield, quality, and zinc content of edible grain fraction synergistically.

## 1 Introduction

Zinc (Zn) is an essential micronutrient for human health, and its deficiency can lead to various adverse effects (Prasad, 2013). Food crops, such as rice, are the main source of dietary Zn (Hall and King, 2022). However, the Zn content in rice is insufficient to meet the body’s requirements (Yang et al., 2021). Zn is also crucial for the growth and development of rice, affecting yield and quality (Naik and Das, 2008; Bana et al., 2021; Tuiwong et al., 2021). Low soil Zn levels (DTPA-Zn < 0.5 mg kg^−1^) contribute to crop Zn deficiency, resulting in negative effects on rice, including poor tillering, reduced root development, low seed-setting rate, and insufficient Zn content in plants (Kumar et al., 2022). Approximately half of the world’s soils are estimated to be deficient or potentially deficient in Zn, posing a significant challenge to public health and the nutritional value of rice (Rehman et al., 2012; Tulchinsky, 2017).

Increasing the Zn content in crops can be achieved through agronomic practices and breeding techniques (Palmgren et al., 2008; Bouis et al., 2011). Adequate application of Zn fertilizer can improve rice yield (Guo et al., 2016), reduce chalkiness, increase protein and amylose content (Ghasal et al., 2016), and enhance Zn uptake and translocation to grains (Ahsin et al., 2020). However, the Zn content in rice grains often falls below the desired levels for human nutrition (Singh et al., 2020). The effectiveness of different Zn fertilizer application methods varies (Elmer and White, 2016), with traditional ionic Zn fertilizers (ZnSO_4_, ZnCl_2_, etc.) being less efficient due to poor migration and absorption by rice roots (Patel et al., 2022). Foliar application shows promise but is affected by environmental factors (Zhang et al., 2017; Soltani et al., 2022). Hence, it is crucial to acquire expertise in the efficient application of Zn fertilizer to enhance rice yield, quality, and Zn content. This knowledge is vital for addressing Zn deficiency in the human body and ensuring food security.

Nanomaterials are widely used in industry (in plant growth), and regulation of its physiological responses is an attempt of recent years (Upadhyaya et al., 2017). Nanomaterials can enter plants through roots, leaves, and other organs, which can improve plant resistance, reduce the degree of plant diseases, and increase the photosynthetic rate of plants. The fertilizer effect of nanomaterials is also reflected in the promotion of plant uptake and utilization, reducing environmental risks (Wang et al., 2018; Bala et al., 2019; Zhang et al., 2022). As a new type of Zn fertilizer, Zn oxide nanoparticles (ZnO NPs) are less affected by soil texture, organic matter, and colloidal material than common Zn fertilizers due to their high-specific surface area (SSA) and surface activity and are easily absorbed and utilized by the rice root system (Zhang et al., 2021). It was found that ZnO NPs application promoted rice yield as well as rice grain Zn content and Zn accumulation better than ionic Zn at the same Zn application rate (Yang et al., 2021). ZnO NPs also improve the salt tolerance of rice pre-growth and further reduces the effect of salt on rice seedling growth vigor, nutritional growth, and flowering and fruit set (Singh et al., 2022). In addition, ZnO NPs are also effective in mitigating the uptake and accumulation of Cd and As in rice seeds (Ma et al., 2020).

Upon application or release into the environment, ZnO NPs undergo rapid dissolution and conversion. Some NPs attach to the crop root surface, while others are taken up by plants as Zn phosphate. Notably, ZnO NPs do not exhibit specific toxicity compared with ZnSO_4_ (Du et al., 2019). Analysis of Zn distribution in the soil reveals that ZnO NPs convert to Zn ions within a short time, penetrating the plant’s tissues and reaching the aboveground parts through the xylem (Lv et al., 2021). The dissolution rate of ZnO NPs is influenced by soil properties, particularly pH and ZnO NPs concentration (Wang et al., 2013). ZnO NPs demonstrate greater efficacy in acidic soils compared with alkaline soils (Sun et al., 2020). They can benefit crop growth and yield at low concentrations but may exhibit toxicity at high concentrations, albeit to a lesser extent than ZnSO_4_. ZnO NPs increase Zn content in crop grains without posing a nano-risk (Du et al., 2019).

Current research on nanomaterials in rice production primarily focuses on their role in promoting rice growth and development, as well as their toxic mechanisms and negative impacts on rice. However, there is limited research on the application methods, appropriate dosages, and their effects on rice yield, quality, and Zn content in the edible portion of the grain. Therefore, this study aimed to investigate the effects of basal application of ZnO NPs on rice yield, quality, and edible grain Zn content at different application rates through field experiments. The objective was to determine the optimal application rate that enhances rice yield, improves quality, and fortifies the edible grain with Zn. The findings of this study will provide a scientific and effective theoretical foundation for the application of ZnO NPs in rice production.

## 2 Materials and methods

### 2.1 Materials

The ZnO NPs used in this study were purchased by the researchers from shanghai Chaowei Nanotechnology Co. (ZnO NPs purity ≥ 99.95%). The ZnO NPs were used as received without any additional purification steps. Under scanning electron microscopy (SEM), the ZnO NPs were spherical with a particle size of 20 nm–50 nm and an SSA of 133.6 m^2^ g^−1^. Conventional urea (46% N), calcium superphosphate (12.5% P_2_O_5_), and potassium chloride (60% K_2_O) were purchased from local fertilizer outlet.

### 2.2 Experimental location and design process

#### 2.2.1 Experimental location

Field trials were conducted in the 2019 and 2020 rice growing seasons at the experimental farm (32°23.4’ N, 119°25.2’ E) of Yangzhou University, Jiangsu Province, China. The soil type was sandy loam with pH 6.76, 1.30 g kg^−1^ total nitrogen, 24.4 g kg^−1^ organic matter, 104.2 mg kg^−1^ alkaline hydrolysable nitrogen, 35.4 mg kg^−1^ Olsen-P, 72.5 mg kg^−1^ exchangeable K, and 0.87 mg kg^−1^ available Zn. The area has a subtropical monsoon climate with a mild climate and abundant rainfall, with an average annual temperature of about 15.2°C and an annual precipitation of about 1020 mm. Meteorological data for the growing season of this crop are shown in **Table 1**.

**Table 1:** Meteorological data of rice growing season.

Month	Temperature (°C)		Rainfall (mm)	
	2019	2020	2019	2020
May	21.41	22.93	38.54	45.68
June	25.20	25.88	113.19	394.89
July	28.32	25.72	91.68	403.72
August	28.58	30.11	151.64	129.85
September	24.30	24.17	56.98	103.09
October	18.97	17.57	6.73	73.00

#### 2.2.2 Design process

The rice variety used in this study was Nanjing 9108, a late-maturing medium *japonica* rice. It was selected by the Institute of Grain Crops, Jiangsu Academy of Agricultural Sciences in Jiangsu, China. Nanjing 9108 is characterized as a multi-spike *japonica* variety. Rice was planted at a density of 12.5 cm × 25 cm with four seedlings per hole. There were six treatments in the field trial: with no ZnO NPs as control (CK); application ZnO NPs 3.75 kg hm^−2^ as T1; application ZnO NPs 7.5 kg hm^−2^ as T2; application ZnO NPs 15 kg hm^−2^ as T3; application ZnO NPs 30 kg hm^−2^ as T4; and application ZnO NPs 60 kg hm^−2^ as T5. The 2019 trial was sown on May 22 and transplanted on June 22, and the 2020 trial was sown on May 19 and transplanted on June 20. The rice was harvested on 18 October 2019 and 16 October 2020, respectively. According to what was observed on the field, the grain‐filling period of rice was 46 days. The N fertilizer in the experiment was 270 kg hm^−2^ and was applied with basal, tillering, and panicle fertilizer at a ratio of 3:3:4 at the relevant growth stage. The potash fertilizer was applied at 270 kg hm^−2^, while the phosphate fertilizer application level was 135 kg hm^−2^. Moreover, the potash fertilizer was applied as 50% basal fertilizer and 50% panicle fertilizer. The phosphate fertilizer was applied entirely as basal fertilizer. During the specific application procedure, the ZnO NPs were uniformly applied by mixing them with quartz sand sieved through a 0.5-mm mesh, according to the predetermined dosage of ZnO NPs. The amount of fertilizer applied and the method of application remained the same for 2 years for each treatment. Treatments were performed on plots of 25 m^2^ (5 m × 5 m), and each treatment was replicated three times. Each plot was separated by a soil monopoly (35 cm wide and 20 cm high) and covered with plastic film. The test plots were flooded after transplanting and the flooding continued until 7 days before maturity. Pests, pathogens, and weeds were controlled with commonly used chemical treatments.

### 2.3 Sampling and data collection

#### 2.3.1 Yield and yield components

At maturity, each plot was harvested manually for 4 m^2^ and weighing the actual yield (14.5% moisture content). Each plot was collected for 1 m^2^ (excluding marginal plants) to examine the number of panicles that was investigated. Thirty representative plants were selected consecutively in each plot to investigate the spikelets per panicle, seed-setting rate and 1,000-grain weight.

#### 2.3.2 Dry matter accumulation

Five consecutive plant samples were collected from each plot at the jointing, heading, and maturity stages, and all samples were separated into leaf, stem (internode plus sheath), and spike tissue. Each sample of rice plants was bagged separately and dried at 105°C for 30 min, then at 80°C to a constant weight.

#### 2.3.3 Rice quality

The rice grains were naturally air-dried, stored at room temperature for 90 days, and then air-selected with a winnowing machine to determine the rice grain quality. The rice quality traits were determined according to China’s National Standard (GB/T17891-2017). A 100-g sample of rice grains was passed through a de-husker for polishing and then separated into broken and unbroken grains. The brown rice rate, milled rice rate, and head rice rate were expressed as percentages of the total (100 g) rice grains. The values for the appearance quality (chalky grain percentage and chalkiness degree) were calculated using a rice appearance quality detector (Hangzhou Wanshen Detection Technology Co., Ltd., Hangzhou, China). The protein and amylose content of milling rice were determined using a 1241 NIR rapid quality analyzer (Infrared 1241 grain analyzer) manufactured by FOSS TECATOR, Sweden. The rice taste meter (Satake Corporation, Higashi-Hiroshima, Japan) was used to automatically measure the taste value of rice. The pasting properties of the rice flour were determined using a rapid viscosity analyzer (RVA, Super3, Newport Scientific, Warriewood, Australia).

#### 2.3.4 Determination of Zn content

Concentration of Zn in stalks, leaves, seeds, hulls, brown rice, paste layer, and milled rice: 0.5 g of the sample was weighed, added to 5 mL of good quality pure nitric acid and subjected to high-temperature digestion in a microwave digester (CEM-MARS 5, USA). The concentration of Zn in the filtrate was determined by plasma emission spectroscopy-atomic absorption (iCAP 6300, USA) after dilution of the digestion solution.

### 2.4 Data analysis


$$\text{Zinc fertilizer utilization rate (\%) = }\frac{\text{Aboveground plant Zn accumulation (g) }}{\text{Pure Zn dosage (g)}}\times 100\%$$

One‐way analysis of variance (ANOVA) was applied using SPSS 20.0 to analyze the data. The LSD test was applied at a 0.05 probability level to establish the significance of discrepancy among each mean.

## 3 Results

### 3.1 Rice yield and its components

The data in **Table 2** depict the influence of ZnO NPs on grain yield and yield component of rice. All 2 years of ZnO NPs increased rice grain yield, with a trend of increasing then decreasing with increasing ZnO NPs. In the 2019, T3 and T4 treatments significantly increased by 4.46%–4.86%, and in the 2020, T1–T5 significantly increased by 3.51%–5.12%, all with the highest yield of rice under T4 treatment.

**Table 2:** Effects of ZnO NPs different application dosage on yield and yield components of *japonica* rice.

Year	Treatment	Panicle (×10^6^ hm^−2^)	Spikelets per panicle	Seed-setting rate (%)	1,000-grain weight (g)	Harvest yield (t hm^−2^)
2019	CK	3.54c	107.82b	90.33c	25.33b	9.87b
	T1	3.73bc	116.16a	90.60c	26.22ab	10.19ab
	T2	3.87ab	116.35a	91.33bc	26.65a	10.24ab
	T3	4.01ab	116.69a	92.00ab	26.63a	10.31a
	T4	4.06a	118.73a	92.62a	27.04a	10.35a
	T5	3.93ab	117.62a	92.89a	26.65a	10.21ab
2020	CK	3.52c	107.82b	89.95b	25.48c	9.96b
	T1	3.86b	113.38ab	90.86ab	25.88bc	10.31a
	T2	3.94ab	113.93ab	91.02ab	25.95abc	10.36a
	T3	4.01ab	114.25ab	91.45ab	26.45ab	10.46a
	T4	4.09a	115.03a	92.18a	26.59a	10.47a
	T5	4.01ab	114.67a	91.86ab	26.33ab	10.33a

Different lowercase letters in the same column indicate significant difference of 5% (results in different years were compared, respectively).

In terms of yield components, the number of panicles increased by 5.37%–14.69% and 9.66%–16.19%, the number of spikelets per panicle increased by 7.74%–10.12% and 5.16%–6.69%, the seed-setting rate increased by 0.3%–2.83% and 1.01%–2.48%, and the 1,000-grain weight increased by 3.51%–6.75% and 1.57%–4.36%, respectively. It is worth noting that, in the 2-year experiment, the number of panicle and the number of spikelets per panicle tended to increase and then decrease with increasing ZnO NPs application in both years, and the best results were obtained under the T4 treatment in both years. In the 2019, the seed-setting rate increased with increasing ZnO NPs application, while in the 2020, the seed-setting rate tended to increase and then decrease with increasing ZnO NPs application, with a significant increase of 2.48% in the T4 treatment; the 1,000-grain weight of the ZnO NPs treatment did not show a regular pattern in the 2019, with a significant increase of 2.13%–6.75% in the T2–T4 treatment compared with the CK, while in the 2020, with increasing ZnO NPs application, the 1,000-grain weight tended to increase and then decrease with increasing ZnO NPs application in the 2020, with the best effect under the T4 treatment with a significant increase of 4.36%.

### 3.2 Dry matter accumulation

Dry matter accumulation in rice is the key to rice yield formation. As shown in **Table 3**, application of ZnO NPs increased dry matter accumulation at the jointing, heading, and maturity, by 10.28%–16.45% and 4.21%–9.41% at the jointing, 11.05%–23.60% and 2.63%–6.32% at the heading, and 9.36%–12.12% and 3.44%–7.00% at the maturity in 2 years, respectively, compared with CK. Different ZnO NPs treatments had no significant effect on the dry matter accumulation of rice at each growth stage. The higher dry matter growth rate at heading stage indicated that ZnO NPs played an important role in rice yield formation during this period. The dry matter accumulation and proportion of rice at different growth stages in **Table 3** also confirmed this hypothesis. The dry matter accumulation and proportion of rice were the highest at heading-maturity stage. The weight of dry matter in a certain period reflected the growth status of the ZnO NPs treatments during that period; the dry matter accumulation and proportion of rice in this period reflect the growth characteristics of rice. Therefore, higher dry matter accumulation at the jointing–heading stage will increase the final yield, which is consistent with the grain yield results. In addition, the dry matter accumulation of rice at the jointing and maturity stages was also higher than that of CK, indicating that ZnO NPs also improved dry matter accumulation in the early and late growth stages of rice.

**Table 3:** Effect of ZnO NPs different application dosage on dry matter accumulation of *japonica* rice.

Year	Treatment	Dry Matter Weight (t hm^−2^)			Sowing - Jointing		Jointing - Heading		Heading - Maturity	
		Jointing	Heading	Maturity	Accumulation (t hm^−2^)	Ratio (%)	Accumulation (t hm^−2^)	Ratio (%)	Accumulation (t hm^−2^)	Ratio (%)
2019	CK	3.89a	10.68a	16.34a	3.89a	23.81a	6.79a	41.55a	5.66a	34.64a
	T1	4.32a	11.86a	17.87a	4.32a	24.17a	7.54a	42.19a	6.01a	33.63a
	T24	4.30a	11.89a	17.91a	4.30a	24.01a	7.59a	42.38a	6.02a	33.61a
	T3	4.29a	11.87a	17.95a	4.29a	23.90a	7.58a	42.23a	6.08a	33.87a
	T4	4.53a	12.20a	18.32a	4.53a	24.73a	7.67a	41.87a	6.12a	33.41a
	T5	4.45a	13.20a	18.26a	4.45a	24.37a	7.69a	42.11a	6.12a	33.52a
2020	CK	4.04a	11.40a	17.72a	4.04a	22.80a	7.36a	41.53a	6.32a	35.67a
	T1	4.21a	11.70a	18.33a	4.21a	22.97a	7.49a	40.86a	6.63a	36.17a
	T2	4.25a	11.85a	18.54a	4.25a	22.92a	7.60a	40.99a	6.69a	36.08a
	T3	4.30a	11.97a	18.74a	4.30a	22.95a	7.67a	40.93a	6.77a	36.13a
	T4	4.42a	12.12a	18.96a	4.42a	23.31a	7.70a	40.61a	6.84a	36.08a
	T5	4.36a	12.05a	18.87a	4.36a	23.11a	7.69a	40.75a	6.82a	36.14a

Different lowercase letters in the same column indicate significant difference of 5% (results in different years were compared, respectively).

### 3.3 Processing and appearance quality

The brown rice rate, the milling rice rate and the head milling rate are important criteria for judging the quality of rice processing. In the 2-year trail, the application of ZnO NPs increased the brown rice rate by 0.65%–1.14% and 0.07%–0.77% and the head milling rate by 2.82%–3.54% and 1.91%–2.99%, respectively. Except for T5 treatment in 2019, other ZnO NPs treatments in the 2-year experiment increased the milling rice rate and were highest in the T1 treatment, while in the 2020 trial, the milling rice rate gradually increased with the increase in the amount of ZnO NPs applied, with the T4–T5 treatments achieving significant levels of improvement compared with CK (as shown in **Table 4**).

**Table 4:** Effects of ZnO NPs different application dosage on processing and appearance qualities of *japonica* rice.

Year	Treatment	Brown rice rate (%)	Milling rice rate (%)	Head milling rate (%)	Chalky grain percentage (%)	Chalkiness degree (%)
2019	CK	84.94c	75.45c	59.26b	57.56a	16.82a
	T1	85.49b	76.89a	60.93ab	53.75ab	16.00a
	T2	85.57ab	76.54ab	60.97ab	52.71ab	18.51a
	T3	85.54b	75.75c	61.17ab	51.61ab	15.64a
	T4	85.91a	75.98bc	61.28ab	47.63b	15.71a
	T5	85.63ab	75.37c	61.36a	50.22b	17.47a
2020	CK	85.23b	75.55c	59.27a	56.81a	20.42a
	T1	85.29b	75.72bc	60.40a	54.20ab	19.56ab
	T2	85.47ab	75.91abc	60.57a	47.07b	15.57b
	T3	85.55ab	76.04abc	60.63a	46.88b	16.23ab
	T4	85.89a	76.21ab	60.90a	52.42ab	17.45ab
	T5	85.70ab	76.31a	61.04a	54.97ab	19.63ab

Different lowercase letters in the same column indicate significant difference of 5% (results in different years were compared, respectively).

Further comparison of the effects of ZnO NPs on rice appearance quality revealed that ZnO NPs application reduced chalky grain percentage and showed a decreasing and then increasing trend with increasing ZnO NPs application, with chalky grain percentage reaching the lowest in treatment T4 in the 2019 trial and the lowest in treatment T3 in the 2020 trial. There was no significant change in chalky grain percentage with increasing ZnO NPs application in the 2019 trial, while in the 2020 trial, chalky grain percentage tended to decrease and then increase with increasing ZnO NPs application, with the lowest chalky grain percentage being achieved in the T2 treatment.

### 3.4 Nutritional and taste quality

In the 2019 trail, compared with CK, the application of ZnO NPs increased the protein content of rice, and the protein content of rice in treatments T3–T5 increased significantly (4.95%–12.79%). In the 2020 trial, rice protein content showed a trend of increasing and then decreasing with increasing ZnO NPs application. In the 2019, the amylose content showed a trend of increasing and then decreasing with the increase in ZnO NPs application, with a significant increase of 6.87%–10.56% compared with CK. In the 2020, the overall trend of ZnO NPs application treatments showed a decrease compared with CK, and none of them reached a significant difference level, with the T5 treatment having the lowest amylose content, with a decrease of 4.24% compared with CK.

Compared with CK, the application of ZnO NPs improved the taste value of rice by 7.30%–10.82% in 2019, with the T2 treatment having the highest taste value; in the 2020, the T2–T4 treatment significantly increased the taste value of rice by 6.80%–8.60% compared with CK, with the T4 treatment having the highest taste value (as shown in **Table 5**). Further analysis of the rice taste value indicators revealed that the increase in taste value was mainly achieved by improving the appearance, stickiness and balance of the rice and reducing the hardness of the rice.

**Table 5:** Effects of ZnO NPs different application dosage on nutrition and eating qualities of *japonica* rice.

Year	Treatment	Protein content (%)	Amylose content (%)	Taste value	Appearance (ce)	Hardness (ss)	Stickiness (ss)	Balance degree(ce)
2019	CK	7.27c	11.36b	71.27b	6.67b	6.70a	7.20b	6.70b
	T1	7.47bc	12.39a	76.47a	7.53a	6.33b	8.17a	7.63a
	T2	7.53bc	12.47a	78.97a	7.97a	6.07b	8.37a	8.00a
	T3	7.63b	12.56a	77.63a	7.67a	6.23b	8.33a	7.77a
	T4	7.63b	12.35a	77.77a	7.77a	6.10b	8.17a	7.80a
	T5	8.20a	12.24a	76.63a	7.53a	6.33b	8.13a	7.60a
2020	CK	7.27a	12.43a	73.97b	7.40b	6.27a	7.50b	7.33b
	T1	7.43a	12.12a	77.67ab	7.80ab	6.20ab	8.27a	7.83ab
	T2	7.63a	12.27a	79.10a	7.97a	6.07ab	8.30a	8.00a
	T3	7.47a	12.35a	79.00a	7.90a	6.07ab	8.43a	8.03a
	T4	7.43a	11.99a	80.33a	8.17a	5.87b	8.40a	8.20a
	T5	7.40a	11.88a	77.40ab	7.70ab	6.20ab	8.20a	7.73ab

Different lowercase letters in the same column indicate significant difference of 5% (results in different years were compared, respectively).

### 3.5 RVA parameters

The RVA profile characteristics are an important indicator of the quality of rice cooking. Compared with CK, the application of ZnO NPs could improve the breakdown, which increased by 17.63%**–**50.59% in 2019 compared with CK; in the 2020, the breakdown significantly increased by 24.52%–31.83% compared with CK. There was a difference in the pattern of change in peak viscosity among the treatments with ZnO NPs, with ZnO NPs increasing peak viscosity in 2019 compared with CK (2.41%–3.39%); in the 2020, compared with the CK, peak viscosity decreased in all the ZnO NPs treatments, except for T4 treatment, with a significant decrease in peak viscosity in T1 3.13%. ZnO NPs application reduced through viscosity by 2.49%–11.86% and 10.62%–14.49% in both years, compared with CK, there is no significant difference in 2019 and a significant difference in 2020. Application of ZnO NPs reduced final viscosity by 0.46%–6.98% and 2.87%–12.96% in both years, respectively. All treatments of ZnO NPs application reduced the setback except for T2 treatment in 2019, and T5 treatment reached a significant difference level compared with CK in 2020. Compared with CK, ZnO NPs application increased the pasting temperature by 0.37%–2.23% and 0.07%–1.96% in both years respectively (as shown in **Table 6**).

**Table 6:** Effects of ZnO NPs different application dosage on RVA profile characteristics of *japonica* rice.

Year	Treatment	Peak viscosity (cP)	Through viscosity (cP)	Breakdown (cP)	Final viscosity (cP)	Setback (cP)	Pasting temperature (°C)
2019	CK	2655.00c	2004.67a	650.33b	2478.00a	-286.33a	73.58b
	T1	2798.33a	1937.67a	860.67ab	2447.00a	-351.33a	74.13ab
	T2	2719.67b	1954.67a	765.00ab	2466.67a	-253.00a	75.22a
	T3	2746.33ab	1767.00a	979.33a	2305.00a	-441.33a	74.93ab
	T4	2740.00ab	1814.00a	926.00ab	2370.33a	-369.67a	73.85ab
	T5	2689.33bc	1772.67a	916.67ab	2342.00a	-347.33a	74.63ab
2020	CK	2708.00ab	1937.33a	770.67b	2430.67a	-277.33a	73.83b
	T1	2623.33c	1656.67b	966.67a	2290.00ab	-333.33a	73.88b
	T2	2690.00abc	1697.33b	992.67a	2361.00a	-329.00a	75.28a
	T3	2680.33abc	1703.33b	977.00a	2236.33ab	-444.00ab	74.12ab
	T4	2747.67a	1731.67b	1016.00a	2289.33ab	-458.33ab	74.45ab
	T5	2648.33bc	1688.67b	959.67a	2115.67b	-532.67b	74.15ab

Different lowercase letters in the same column indicate significant difference of 5% (results in different years were compared, respectively).

### 3.6 Aboveground Zn accumulation in rice

As presented in **Table 7**, the Zn content of aboveground rice plants treated with ZnO NPs increased with the increase of ZnO NPs application. In the 2019, Zn content in rice stalks and leaves of T3–T5 treatments were significantly increased by 64.95%–92.48% and 50.20%–116.61%, respectively, compared with CK; in the 2020, the Zn content in rice stems treated with T4 and T5 was significantly increased by 90.28%–128.02% compared with CK, and the Zn content in rice leaves treated with T4 and T5 was significantly increased by 112.86%–156.79%. The Zn content in rice grains increased with increasing ZnO NPs application, with significant increases of 16.67%–35.35% and 12.49%–30.27% compared with CK in both years, respectively. Zn accumulation in aboveground rice plants also increased with the increase of ZnO NPs application, compared with CK, Zn accumulation in above ground plants was significantly increased by 39.26%–124.00% and 33.80%–118.38% in both years by the application of ZnO NPs, respectively.

**Table 7:** Effect of ZnO NPs different application dosage on Zn accumulation in aboveground plants of *japonica* rice.

Year	Treatment	Stem zinc content (mg kg^−1^)	Leaf zinc content (mg kg^−1^)	Grain zinc content (mg kg^−1^)	Stem zinc accumulation (g hm^−2^)	Blade zinc accumulation (g hm^−2^)	Grain zinc accumulation (g hm^−2^)	Aboveground part zinc accumulation (g hm^−2^)
2019	CK	64.13c	49.68c	16.86f	345.07e	99.63f	163.80d	608.50e
	T1	74.09bc	64.60bc	19.67e	509.29d	136.36e	201.78c	847.42d
	T2	89.56abc	74.62abc	20.95d	615.99c	156.41d	216.10b	988.50c
	T3	105.78ab	85.82ab	21.79c	719.49b	175.53c	228.54ab	1123.55b
	T4	113.35a	97.50ab	22.47b	826.56a	196.99b	237.49a	1261.04a
	T5	123.44a	107.61a	22.82a	915.11a	217.16a	230.78a	1363.05a
2020	CK	54.71c	42.61c	17.54e	360.92f	104.29e	168.07d	633.28f
	T1	72.41bc	64.61bc	19.73d	506.64e	137.22d	203.46c	847.32e
	T2	83.33abc	69.33abc	21.08c	615.30d	157.87c	218.27b	991.44d
	T3	95.57abc	78.69abc	21.87bc	710.78c	175.20bc	228.66a	1114.63c
	T4	104.10ab	90.70ab	22.43ab	807.71b	194.45b	234.84a	1237.00b
	T5	124.75a	109.42a	22.85a	925.25a	221.67a	236.02a	1382.94a

Different lowercase letters in the same column indicate significant difference of 5% (results in different years were compared, respectively).

### 3.7 Zn content and proportion of Zn content of each part of the grain

The application of ZnO NPs all significantly increased the Zn content in the milled rice, brown rice, grain coat, and aleurone layer. Compared with CK, the application of ZnO NPs significantly increased the Zn content of milled rice by 20.46%–41.09% and 18.11%–38.84%, respectively. In the 2019 trail, Zn content in milled rice showed an increasing and then decreasing trend with increasing ZnO NPs application, with the highest Zn content in T4 treatment, while 2020 did not show a clear pattern with increasing ZnO NPs application. Compared with CK, the Zn content of brown rice increased by 25.78%–48.30% and 20.86%–42.00% in both years, respectively, and the Zn content of brown rice and grain coat and aleurone layer showed an increasing and then decreasing trend with the increase of ZnO NPs application, and reached the maximum in T4 treatment. The Zn content increasing of brown rice was remarkably lower than the vegetative organs, indicating that more Zn was sediment in the vegetative organs and not efficiently positioned in rice grains. The Zn content of rice hull did not show a significant pattern of change, and compared with CK, all the treatments with ZnO NPs application increased the Zn content of rice hull, except for T1 and T4 treatments in 2020 (as shown in **Table 8**).

**Table 8:** Effect of ZnO NPs different application dosage on Zn content in *japonica* rice.

Year	Treatment	Milled rice (mg kg^−1^)	Grain coat and aleurone layer (mg kg^−1^)	Brown rice (mg kg^−1^)	Rice hull (mg kg^−1^)
2019	CK	11.39d	32.81f	13.81d	32.25b
	T1	13.72c	44.78e	17.37c	33.52b
	T2	13.90c	47.07d	17.55c	41.01a
	T3	15.33b	48.75c	19.07b	38.24a
	T4	16.07a	54.63a	20.48a	34.67b
	T5	15.79ab	50.53b	19.92a	40.74a
2020	CK	10.66c	32.56f	14.19d	36.82bc
	T1	12.59b	44.45e	17.15c	35.09c
	T2	14.20a	46.73d	17.45c	42.42a
	T3	14.70a	48.40c	19.01b	38.63b
	T4	14.62a	54.24a	20.15a	36.02bc
	T5	14.80a	50.17b	19.72ab	41.96a

Different lowercase letters in the same column indicate significant difference of 5% (results in different years were compared, respectively).

**Figure 1:**
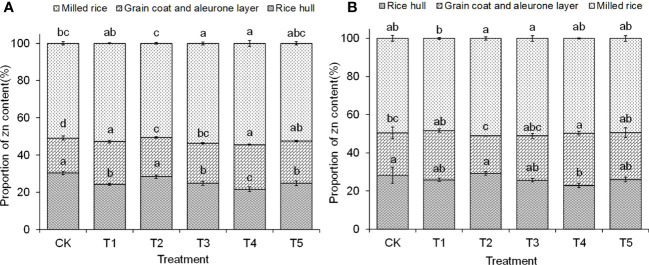
Effects of ZnO NPs dosages on the ratio of zinc content in different parts of *japonica* rice grain. **(A)**, 2019; **(B)**, 2020. Different lowercase letters in the same column indicate significant difference of 5% (results in different years were compared, respectively).

In the 2-year trial, except for the T2 treatment, the application of ZnO NPs was able to reduce the proportion of Zn in the rice hull and all reached a significant level of difference. Except for the T2 treatment in 2020, all applied ZnO NPs treatments increased the proportion of grain and aleurone layer to grain Zn to varying degrees. Except for the T2 treatment in 2019, all treatments with ZnO NPs application significantly increased the proportion of milled rice to grain Zn (as shown in **Figure 1**). This result may be due to the fact that ZnO NPS facilitated the transfer of Zn to the milled rice, which is conducive to promoting greater human intake of Zn from rice.

### 3.8 Zn fertilizer utilization

**Figure 2:**
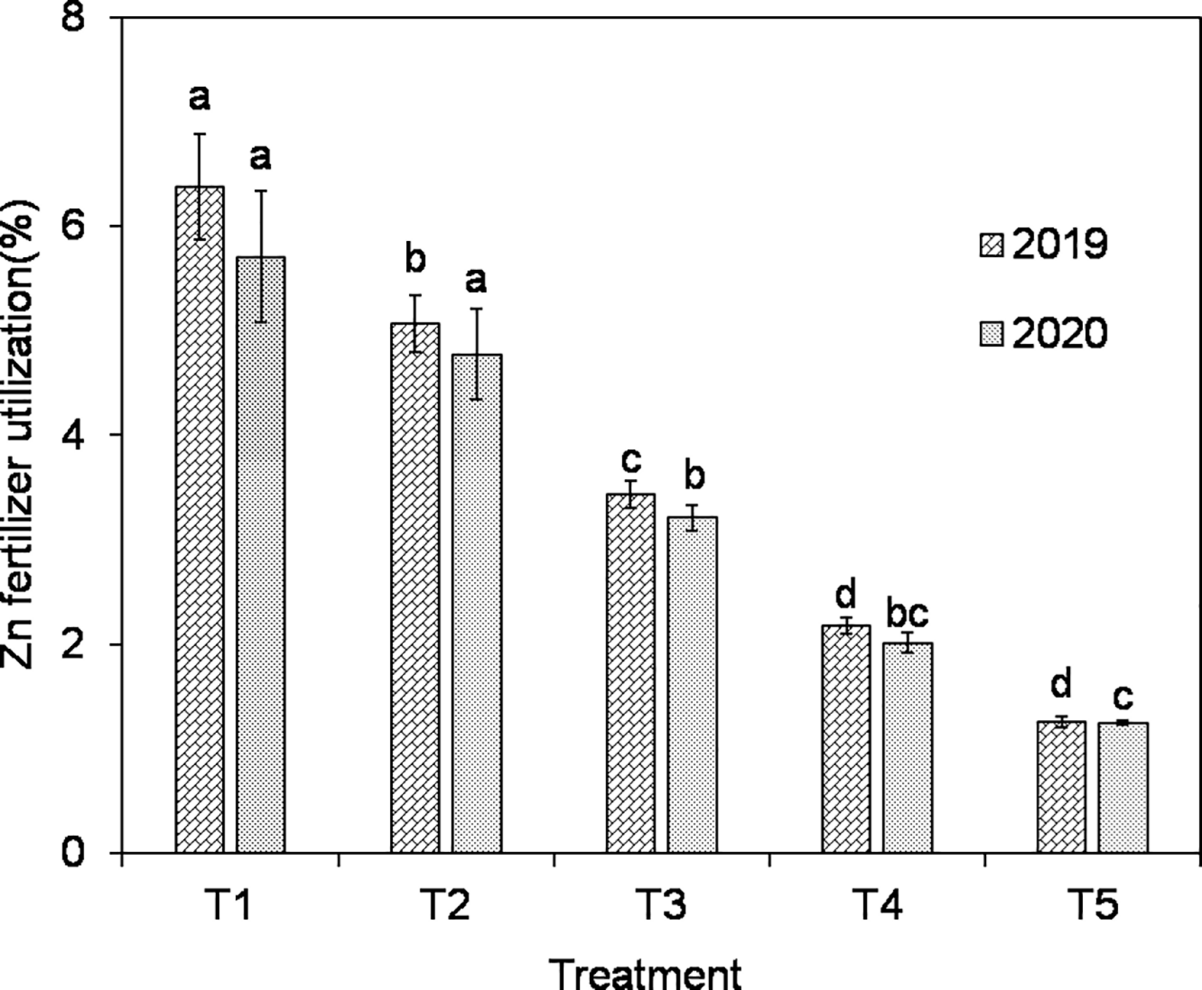
Effects of ZnO NPs dosages on utilization rate of Zn fertilizer in mature stage of *japonica* rice. Different lowercase letters in the same column indicate significant difference of 5% (results in different years were compared, respectively).

In the 2-year trial, Zn fertilizer utilization decreased with increasing ZnO NPs application, with all treatments showing a significant decrease in the 2019 except for the T5 treatment, while in the 2020, the T3 treatment showed a significant decrease and the remaining treatments did not show a significant decrease and showed a slow decrease (as shown in **Figure 2**).

## 4 Discussion

### 4.1 Grain yield

In the present study, the application of ZnO NPs resulted in increases in rice grain yield of 3.24%–4.86% and 3.51%–5.12% in the 2-year trials compared with the control (CK). The tested soil belonged to the effective area of Zn fertilizer according to the Zn fertilizer application classification in China (Liu, 1994). Supplementing ZnO NPs fertilizer on Zn-deficient soils can enhance crop yields. Previous research by Guo et al. (2016) also demonstrated that Zn fertilizer application significantly improves rice yield components. In our study, ZnO NPs application on potentially Zn-deficient soils increased rice yield by promoting panicle and spikelet development, leading to a larger population of glumes in rice. Higher dry matter accumulation is associated with higher rice yields, as the formation of rice yield is primarily a process of dry matter production and distribution (Cheng et al., 2022). Our results indicated that ZnO NPs applications at the jointing, heading, and maturity stages enhanced dry matter accumulation, suggesting the positive impact of ZnO NPs on final yield. Zn plays a crucial role in various enzymatic processes within plants, including chlorophyll synthesis and carbohydrate metabolism. By increasing the application of Zn fertilizer, photosynthesis and the efficiency of photosynthetic assimilate synthesis in rice are enhanced. This, in turn, promotes the accumulation of dry matter in rice, which is essential for achieving high yields. There was a significant positive correlation between rice yield and Zn application within a certain range, but excessive Zn application can have toxic effects on rice and may not be beneficial for yield increase (Bana et al., 2021; Tuiwong et al., 2021). In our experiment, rice yield increased and then decreased with increasing ZnO NPs application. The highest rice yield was achieved at an application rate of 30 kg hm^−2^ of ZnO NPs and further increases in ZnO NPs led to a decrease in yield, with the lowest yield observed at an application rate of 60 kg hm^−2^. However, even at the highest application rate of ZnO NPs, the yield remained higher than when 3.75 kg hm^−2^ of ZnO NPs was applied. This could be attributed to the active adsorption capacity of ZnO NPs, which promotes the uptake of elemental phosphorus by the plant through its interaction with phosphate ions, while also reducing the toxic effects of ZnO NPs on rice (Akram et al., 2020).

The price of ZnO NPs on the market is ¥120 kg^−1^, compared with the application of ZnO NPs 3.75 kg hm^−2^, when applying 60 kg hm^−2^, the increase in income in the 2-year trial is only ¥56 (the market price of rice is calculated as ¥2.8 kg^−1^), but the application of ZnO NPs fertilizer costs ¥6,750 more, in terms of economic efficiency, 60 kg hm^−2^ of ZnO NPs is not the most appropriate amount. As a new type of fertilizer, ZnO NPs can greatly improve the ecological benefits of the soil, enhance the water and fertilizer retention capacity of the soil, and promote sustainable crop production. In terms of price, ordinary ionic Zn costs ¥10–30 kg^−1^ and ZnO NPs is four to 12 times more expensive; ZnO NPs does not have an advantage. However, by reducing the purity of ZnO NPs in agricultural production, finding suitable application rates for ZnO NPs, or achieving large-scale production, ZnO NPs can be used to achieve higher yield and ecological benefits at a lower capital investment.

### 4.2 Grain quality

The quality of rice encompasses various aspects such as processing quality, appearance quality, nutritional quality, taste quality, and cooking quality. Previous research has demonstrated that the application of Zn fertilizer can enhance the processing quality of rice by increasing the percentage of brown rice and milled rice (Zhen et al., 2012). In line with these findings, the results of our study indicate that the application of ZnO NPs leads to improvements in the brown rice rate and milling rice rate, with some impact on the head milling rate as well. This improvement can be attributed to the promotion of grain filling and a reduction in grain breakage during the milling process, thus enhancing the overall processing quality of the rice. Moreover, the application of ZnO NPs enhances the assimilation of photosynthesis products in rice, facilitating their transfer to the kernels (Asif et al., 2005). This leads to a reduction in kernel breakage during the milling process, thereby improving the processing quality of rice. Additionally, the increased degree of grain assimilation contributes to improved appearance quality of the rice.

Chalkiness in rice grains, caused by uneven assimilate distribution, affect appearance quality (Wang et al., 2021). In our study, ZnO NPs application in 2019 did not show a significant trend in chalkiness. However, in 2020, increasing ZnO NPs application led to a decrease followed by an increase in chalkiness. The best appearance quality was observed at a ZnO NPs application rate of 15 kg hm^−2^, while higher rates resulted in a decline. Zn ion concentration affects assimilate transport efficiency, with moderate Zn fertilization enhancing photosynthesis and assimilate synthesis (Tondey et al., 2022). Conventional Zn fertilizers may not be effective in Zn-deficient soils due to rapid immobilization (Baddar and Unrine, 2021). In contrast, ZnO NPs slowly dissolve, acting as a sustained Zn supply, promoting plant growth and grain filling (Zhang et al., 2018). Zn enrichment improves grain assimilate filling, but excessive Zn can be toxic, reducing protein content and disrupting photosynthesis (Li et al., 2022a). Zn fertilizer application within a certain range increases protein and starch accumulation in grains (Singh et al., 2021). In 2019, ZnO NPs increased rice protein content, while in 2020, it showed an increasing-then-decreasing trend. Amylose content followed a similar pattern in 2019, with an overall decreasing trend in 2020. Nitrogen-Zn interactions and rainfall may influence nitrogen metabolism and affect protein and amylose content (Liang et al., 2015; Kuroha and Ashikari, 2020). Zn treatment may also modulate genes and enzymes involved in amylose synthesis (Zhao et al., 2022). Further investigation is needed to determine the exact causes.

ZnO NPs application had positive effects on the cooking and eating quality of rice, resulting in several improvements. The treated rice exhibited enhanced taste, appearance, stickiness, and balance, while its hardness was reduced (Ge et al., 2022). These quality enhancements can be attributed to the role of Zn as a cofactor for flavor enzymes and an activator of these enzymes. At specific temperatures, Zn and other trace element ions activate flavor enzymes, leading to the breakdown of sugars, fats, and proteins in rice. This breakdown process increases the presence of fatty acids, amino acids, aldehydes, ketones, vinegars, and other flavor compounds in the rice, ultimately improving its taste (Suganya et al., 2020). In comparison with traditional ionic Zn, ZnO NPs more effectively increase the Zn concentration in the grains, thereby promoting the activation of flavor enzymes and enhancing the overall taste value of rice.

The characteristics of the RVA spectrum, such as breakdown and setback, are closely associated with the taste quality of steamed rice (Xuan et al., 2020; Xu et al., 2021). Generally, rice with better taste quality tends to exhibit higher peak viscosities, lower breakdown, and lower setback values. Studies have shown that a decrease in amylose content promotes water absorption and swelling of rice, resulting in enhanced stickiness and increased breakdown, which ultimately improves the palatability of rice (Derycke et al., 2005). In this study, the application of ZnO NPs in 2019 led to enhanced peak rice viscosity, increased breakdown, and higher pasting temperature. All treatments, except for the application of Zn at a rate of 7.5 kg hm^−2^, reduced rice setback. In 2020, the application of ZnO NPs increased rice breakdown and pasting temperature while reducing rice setback. However, there was no significant pattern of change observed in peak viscosity. These variations in results between the two years may be attributed to environmental factors. Heavy rainfall in 2020 reduced the plants’ photosynthetic capacity, resulting in a limited availability of sugar sources. This decrease in sugar sources could have inhibited starch expansion, leading to a reduction in amylose content. Consequently, the lack of sugar sources and limited starch expansion may have contributed to the absence of significant changes in peak rice viscosity (Ai et al., 2023).

### 4.3 Grain Zn content

The additional application of Zn fertilizer has been observed to increase the Zn content and accumulation in plants, indicating its influence on Zn translocation and distribution within the plant (Yang et al., 2021; Zhang et al., 2021). Studies have shown that the Zn content and accumulation in aboveground rice plants increased with the application of ZnO NPs (Li et al., 2022b). Even when the external Zn levels reached toxic levels for the plants, rice plants continued to uptake and accumulate exogenous Zn uncontrollably. It has been established that externally added Zn fertilizer serves as the main source of Zn in Zn-deficient or potentially Zn-deficient soils (Montalvo et al., 2016). When applied as a novel Zn fertilizer, ZnO NPs not only have the potential to be directly absorbed by rice roots but also gradually release Zn ions, thereby supporting the growth and development of rice (Du et al., 2019).

Zn content and accumulation in different parts of the rice plant increased with Zn application, with stalks showing higher levels compared with leaves and grains (Paramesh et al., 2020; Zhang et al., 2021). In our study, Zn content followed the order: stalk > leaf > grain, and Zn accumulation followed the order: stalk > grain > leaf, with increasing ZnO NPs application. The enrichment of Zn in stalks can be attributed to their role as the primary pathway for Zn transport to the grains. ZnO NPs application may affect the translocation process, as they can interact with organic acids in the roots and become fixed in the roots (Palmgren et al., 2008). This interaction influences the availability of Zn from ZnO NPs as a fertilizer. These findings highlight the potential impact of ZnO NPs on Zn distribution within the rice plant, particularly in the stalks. Understanding the mechanisms involved is essential for optimizing the use of ZnO NPs as a fertilizer in rice cultivation.

**Figure 3:**
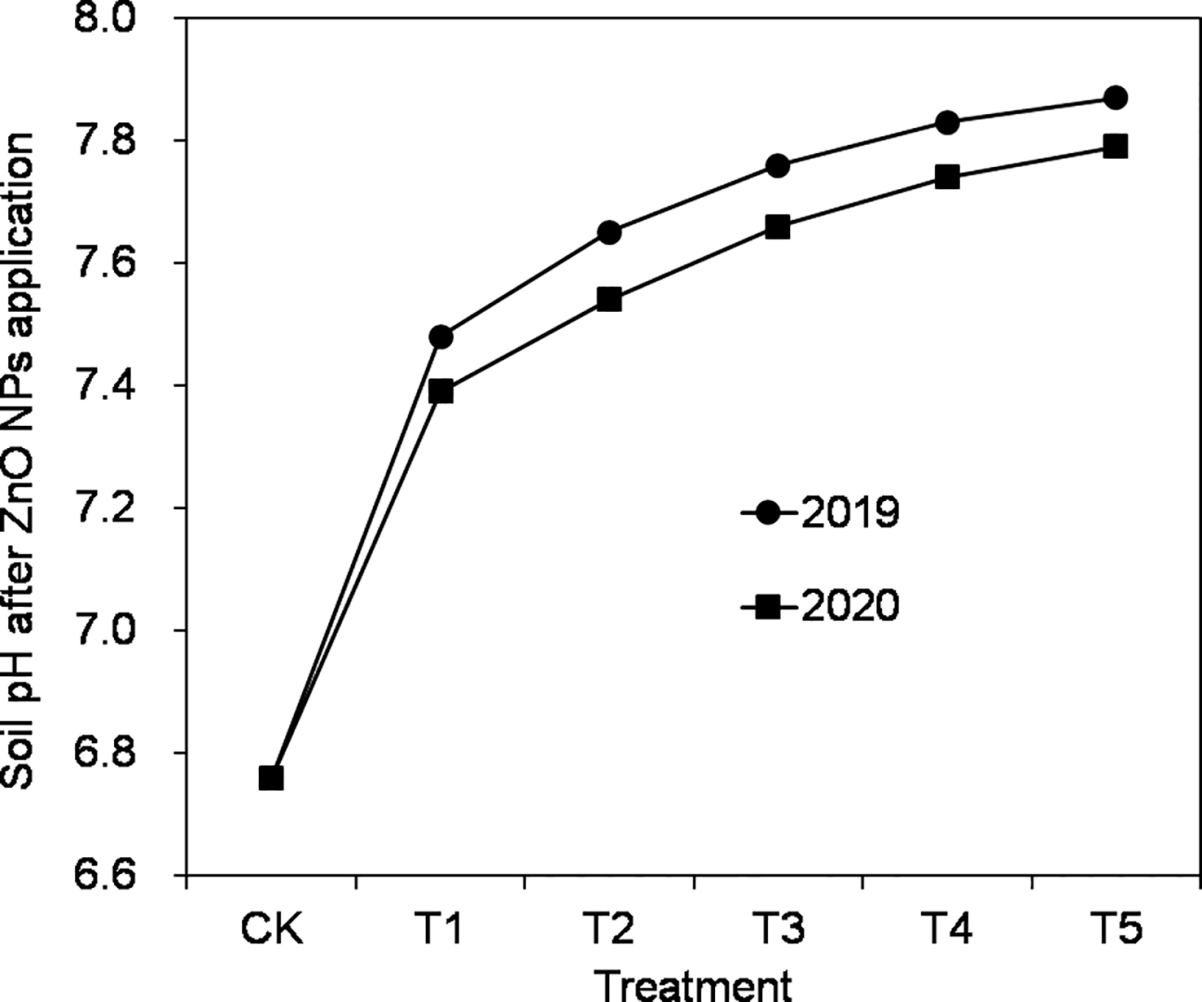
Effect of ZnO NPs application dosages on soil pH.

As the amount of ZnO NPs applied increased, there was a gradual decrease in the utilization of Zn fertilizer. This decline can be attributed to several factors. First, the application of higher levels of ZnO NPs inhibited plant biomass accumulation, and there was a correlation between Zn accumulation and biomass presence (Repkina et al., 2023). This indicates that, as the concentration of ZnO NPs increased, the ability of the plants to effectively utilize the Zn fertilizer decreased. Additionally, it was observed that the ZnO NPs applied to the soil rapidly converted into a form that was not readily available to the plant root system, leading to the loss of Zn fertilizer and making it difficult for rice to absorb and utilize the Zn to its maximum extent (Zhang et al., 2018). Moreover, the neutral pH of the test soil (pH = 6.76) in this study could have reduced the effectiveness of ZnO NPs. The 2-year trial consistently showed an increase in soil pH with increasing ZnO NPs application (as shown in **Figure 3**). This increase in soil pH can be attributed to the dissolution of Zn ions from ZnO NPs, which raises the pH and subsequently hampers Zn fertilizer utilization (Sheteiwy et al., 2021). Furthermore, the transport process of Zn in the soil involves complex interactions between the soil, plants, and seeds, influenced by factors such as root secretions and organic acids produced by the plants (Palmgren et al., 2008). Despite the increased uptake of Zn by the plants, the overall utilization of Zn fertilizer showed a gradual decrease compared with the increase in ZnO NPs application.

Increasing the Zn content in milling rice is important for consumption. The application of ZnO NPs in this study significantly increased the Zn content in milling rice, showing a range of 20.46%–41.09% and 18.11%–38.84% higher compared with the control group in the 2-year trial. The most effective increase in Zn content in milling rice was observed at 30 and 60 kg hm^-2^ of ZnO NPs. After being applied to the soil, ZnO NPs were partially absorbed directly by the rice plant roots. During root uptake and transportation within the plant, the NPs were broken down by organic acids present in the plant, and the released Zn was utilized in the form of soluble Zn^2+^ or organic Zn (Dimkpa et al., 2013). Another portion of ZnO NPs dissolved in the soil due to acidic substances and combined with phosphate ions, resulting in Zn phosphate that was then used by rice plants. Amino acids, as organic molecules, can enhance plant response and absorption of Zn after reacting with it, thereby increasing the Zn content in milled rice (Xiao et al., 2022). Previous studies (Dimkpa et al., 2013; Wang et al., 2013) have shown that when ZnO NPs are applied to the soil, plants take up Zn in the form of soluble Zn^2+^ rather than as ZnO NPs. This indicates that the application of ZnO NPs increases the concentration of Zn in rice grains without posing risks to human consumption or health.

Phytic acid, such as inositol hexakisphosphate, can limit the bioavailability of Zn and hinder its uptake by humans (Jaksomsak et al., 2018). In rice grains, Zn is unevenly distributed, with higher levels found in the embryo and dextrin layer, and lower levels in the endosperm, leading to Zn enrichment in non-edible parts such as the rice hull and bran (Zhang et al., 2012; Castro-Alba et al., 2019). In this study, the application of ZnO NPs significantly increased the Zn content in all parts of the grain, particularly in brown rice and milled rice, while reducing the proportion of Zn in the rice hull. However, a substantial portion of Zn still remained in non-edible parts such as the grain coat and aleurone layer, accounting for over 45% of the total Zn content. Despite this, the ZnO NPs treatment improved rice processing quality, increasing the proportion of brown rice and milled rice, which may contribute to improved Zn delivery and human health benefits.

## 5 Conclusion

In this study, field experiments were conducted to evaluate the effects of basal application of ZnO NPs at different rates on rice yield, quality, and edible grain Zn content to determine the optimal application rate with potential for application. The findings of this study provide strong evidence for the positive effects of ZnO NPs application on rice production, quality, and Zn content. Applying ZnO NPs at rates between 7.5 kg and 30 kg hm^−2^ resulted in increased rice yield, improved grain quality, and enhanced Zn accumulation in the grains. The application of ZnO NPs led to increase the number of panicles, spikelets per panicle, seed-setting rate and 1,000-grain weight, all of which contributed to higher rice yields. Additionally, ZnO NPs application promoted dry matter accumulation throughout the growth period of rice, further supporting increased yields. The use of ZnO NPs also had noticeable benefits on rice processing, appearance, nutrition, taste, and steaming quality. The increased Zn content in the grain, brown rice, and milled rice indicated the effectiveness of ZnO NPs in enhancing the nutritional value of rice. However, it is essential to consider the soil environment when applying ZnO NPs in the field, as high concentrations of ZnO NPs in the rhizosphere may have adverse effects on rice roots. Further research is required to determine the optimal application methods to ensure the safe and effective use of ZnO NPs for different rice varieties and soil conditions. Overall, the results highlight the potential of ZnO NPs as a promising trace element fertilizer for achieving high-yielding, high-quality, and Zn-enriched rice production.

## Data availability statement

The original contributions presented in the study are included in the article/supplementary material. Further inquiries can be directed to the corresponding author.

## Author contributions

KM and XY: experimental design, data analysis and edited and improved the manuscript. QW, CD, and RW: participate in data collection, validation and investigation. SY and YY: writing the original draft and data curation. HPZ and HCZ: validation, project management, funding acquisition and supervision. All authors contributed to the article and approved the submitted version.
